# Supplementary material for: Pre-Emptive Antimicrobial Locks Decrease Long-Term Catheter-Related Bloodstream Infections in Hemodialysis Patients
Source: Antibiotics (Basel). 2022 Nov 24;11(12):1692. doi: 10.3390/antibiotics11121692 (PMC9774347; doi:10.3390/antibiotics11121692)
Supplement: Supplementary file 1 [file antibiotics-11-01692-s001.zip › antibiotics-2036205-supplementary.pdf]

Supplementary material

Pre-Emptive Antimicrobial Locks Decrease Long-Term Catheter-Related Bloodstream Infections in Hemodialysis Patients

Andres Blanco-Di Matteo [1], Nuria Garcia-Fernandez [2], Aitziber Aguinaga Pérez [3,4], Francisco Carmona-Torre [1,4], Amaya C. Oteiza [5], Jose Leiva [4,5] and Jose Luis Del Pozo [1,4,5,*]

1 Infectious Diseases Division, Clinica Universidad de Navarra, 31008 Pamplona, Spain
2 Nephrology Department, Clinica Universidad de Navarra, 31008 Pamplona, Spain
3 Clinical Microbiology Department, Hospital Universitario de Navarra, 31008 Pamplona, Spain
4 Navarra Health Research Institute-IdiSNA, 31008 Pamplona, Spain
5 Clinical Microbiology Department, Clinica Universidad de Navarra, 31008 Pamplona, Spain
* Correspondence: jdelpozo@unav.es

Table S1. Detailed isolation of quantitative blood cultures.

Bacteria	N
Staphylococcus epidermidis	168
Staphylococcus haemolyticus	15
Kocuria kristinae	12
Staphylococcus warneri	11
Staphylococcus hominis	6
Enterococcus faecalis	6
Enterococcus faecium	6
Staphylococcus aureus	6
Staphylococcus capitis	6
Corynebacterium afermentans	4
Klebsiella pneumoniae	4
Escherichia coli	3
Micrococcus luteus	2
Klebsiella oxytoca	2
Sphingomonas paucimobilis	2
Staphylococcus saprophyticus	2
Streptococcus bovis	2
Bacillus spp	2
Acinetobacter ursingii	1
Corynebacterium tuberculostearicum	1
Corynebacterium urealyticum	1
Cutibacterium acnes	1
Pseudomonas aeruginosa	1
Pseudomonas putida	1
Streptococcus gordonii	1
Streptococcus pyogenes	1
Negative	2762
**Total**	**3029**
